# Adverse drug reaction monitoring in psychiatry out-patient department of an Indian teaching hospital

**DOI:** 10.4103/0253-7613.75664

**Published:** 2011-02

**Authors:** Gairik Sengupta, Subhrojyoti Bhowmick, Avijit Hazra, Ananya Datta, Musfikur Rahaman

**Affiliations:** Department of Pharmacology, Institute of Postgraduate Medical Education & Research (IPGME&R), 244B, Acharya J. C. Bose Road, Kolkata - 700 020, India

**Keywords:** Adverse drug reactions, pharmacovigilance, psychiatry, psychotropic drugs

## Abstract

**Objectives:**

Adverse drug reactions (ADRs) to psychotropic agents are common and can lead to noncompliance or even discontinuation of therapy. There is paucity of such data in the Indian context. We deemed it worthwhile to assess the suspected ADR profile of psychotropic drugs in an ambulatory setting in a public teaching hospital in Kolkata.

**Materials and Methods:**

A longitudinal observational study was conducted in the outpatient department (OPD) of the concerned psychiatry unit. Twenty consecutive patients per day, irrespective of their psychiatric diagnosis, were screened for suspected ADRs, 2 days in a week, over 15 months. Adverse event history, medication history and other relevant details were captured in a format as adopted in the Indian National Pharmacovigilance Programme. Causality was assessed by criteria of World Health Organization-Uppsala Monitoring Center (WHO-UPC).

**Results:**

We screened 2000 patients (68.69% males, median age 34.4 years), of whom 429 were suspected of having at least one ADR; 84 cases had insufficient evidence about causality (WHO-UMC causality status “unlikely”) and were excluded from further analysis. Thus, 17.25% (95% confidence interval: 15.59-18.91%) of our study population reported ADRs with at least “possible” causality. Of 352 events recorded, 327 (92.90%) were “probable” and the rest “possible”. None was labeled “certain” as rechallenge was not performed. Patients received a median of 3.2 psychotropic drugs each. Thirty-three different kinds of ADRs were noted, including tremor (19.60%), weight gain (15.34%) and constipation (14.49%). Among the incriminated drugs, antipsychotics represented the majority (57.10%), with olanzapine topping the list.

**Conclusions:**

This study offers a representative profile of ADRs to be expected in psychiatry out-patients in an Indian public hospital. Establishment of a psychotropic drug ADR database can be a worthy long-term goal in the Indian context.

## Introduction

Psychotropic drugs are plentiful in number and their use is increasing day by day. These drugs are capable of causing a number of adverse drug reactions (ADR),[1,2] some of which may be fatal.[3] ADRs associated with psychotropic drugs can lead to noncompliance, and at times discontinuation of therapy.[4] Pharmacovigilance in psychiatry units can play a vital role in detecting ADRs and alerting physicians to the possibility and circumstances of such events, thereby protecting the user population from avoidable harm.[5] In India, pharmacovigilance activities are still in nascent stage and there are few reports available on the ADR profile of medicines in general and psychotropic agents in particular. This prompted us to evaluate the ADR profile of psychotropic drugs used by ambulatory patients in a teaching hospital.

## Materials and Methods

A longitudinal observational study was undertaken in the psychiatry out-patient department (OPD) of Bangur Institute of Neuroscience and Psychiatry, Kolkata, between January 2007 and March 2008. This hospital caters mostly to non-affluent sections of the community, like other public hospitals in Kolkata. It was part of ongoing pharmacovigilance activity at the Institute which had the necessary administrative and Institutional Ethics Committee clearance.

Twenty consecutive patients per day, irrespective of their psychiatric diagnosis, were screened for suspected ADRs, for two fixed days in a week, barring public holidays. The screening was carried out by three psychiatry and three pharmacology residents trained in the psychiatry department for interviewing mental patients. Subjects and their accompanying family members were interviewed, and past prescriptions and case notes, where available, were reviewed. A senior psychiatrist was available for consultation in the event of any difficulty. Patients who were known substance abusers and psychotic subjects not accompanied by a family caregiver were not included in the study. Patient details (age, sex, body weight), adverse event history, history of medication suspected of having caused the ADR, and details of concomitant medication use were recorded in the format followed in the Indian National Pharmacovigilance Programme.[6] It was decided a priori that events of only mild severity along with considerable confusion in differentiating from disease symptomatology will not be counted as suspected ADRs.

Causality of the event was assessed by World Health Organization-Uppsala Monitoring Centre (WHO-UMC) criteria.[7] This analysis was conducted by two pharmacology residents in consultation with a senior pharmacologist. Suspected ADRs with causality status less than “possible” were not considered for further analysis.

## Results

A total of 2000 patients were screened for the study, of whom 429 (21.45%) were suspected of having at least one ADR. On causality assessment, 84 of these 429 cases (19.58%) were considered to have insufficient evidence about causality (WHO-UMC causality status “unlikely”) and they were excluded from further analysis. From the remaining 345 subjects, 352 ADRs were tabulated. Thus, 17.25% (95% confidence interval: 15.59–18.91%) of our study subjects reported ADRs with at least “possible” causality.

Males represented 68.69% of the cases. Notably, on an average day, about 60% of the patients attending the concerned OPD were males. The median (interquartile range) age of the subjects was 34.4 (14) years. Bipolar affective disorder (27%) was the commonest clinical diagnosis among these cases, followed by schizophrenia (24%) and depression (22%). Patients received 3.2 (1.5) [median (interquartile range)] psychotropic drugs per prescription. A few subjects were taking concomitant medicines for other disorders such as dyspepsia and hypertension, started before their psychotropic medication, or OTC medicines casually for minor ailments like cough and cold. The drug history was taken very carefully in such cases before attributing suspected ADRs to the psychotropic medicines concerned.

Thirty-three different kinds of treatment emergent ADRs were encountered in the patients as listed in listed in Table 1. Tremor (19.60%) was the commonest ADR noted, closely followed by weight gain (of 5 kg or more over baseline weight; 15.34%) and constipation (14.49%). Causality assessment revealed that 327 ADRs (92.90%) belonged to “probable” category, whereas 25 (7.10%) were of “possible” type according to the WHO-UMC scale. No case could be labeled “certain”, as rechallenge was not attempted by the attending psychiatrist, once a drug was withdrawn.

**Table 1:** Spectrum of suspected adverse drug reactions (ADRs) noted among 345 patients

*Type of ADR*	*No. (percentage of all ADRs; n = 352)*
Tremor	69 (19.60)
Weight gain	54 (15.34)
Constipation	51 (14.49)
Dyspepsia	35 (9.94)
Extrapyramidal reactions	29 (8.24)
Amenorrhea	22 (6.25)
Anorexia	16 (4.54)
Alopecia	12 (3.41)
Sedation	9 (2.55)
Sexual dysfunction	4 (1.14)
Curling of scalp hair	3 (0.85)
Headache	3 (0.85)
Hypersalivation	3 (0.85)
Hypotension (postural)	3 (0.85)
Impaired liver function (liver enzymes over twice	3 (0.85)
normal)	
Increased appetite	3 (0.85)
Increased nightmares	3 (0.85)
Insomnia	3 (0.85)
Edema (pedal)	3 (0.85)
Agitation and restlessness	2 (0.57)
Hypothyroidism	2 (0.57)
Impaired glucose tolerance	2 (0.57)
Leukocytosis	2 (0.57)
Leukopenia	2 (0.57)
Polyuria	2 (0.57)
Rashes	2 (0.57)
Seizure precipitation	2 (0.57)
Tachycardia	2 (0.57)
Vertigo	2 (0.57)
Diabetes mellitus, impaired accommodation for near vision, Rise of BP, retention of urine	1 (0.28) each

This list excludes events in 84 subjects where causality status was less than “possible”

Among the drugs incriminated, antipsychotics (typical and atypical) were the commonest group of agents causing ADRs (57.10%), followed by selective serotonin reuptake inhibitors (17.61%) and lithium (13.35%), as has been summarized in Table 2. Olanzapine was the commonest single drug (31.82%) incriminated followed by haloperidol (19.03%).

**Table 2:** Drugs responsible for 352 adverse drug reactions noted among 345 patients

*Drugs responsible for 352 adverse drug reactions noted among 345 patients*	*No. (Percentage of all ADRs)*
Olanzapine	112 (31.82)
Haloperidol	67 (19.03)
Selective serotonin reuptake inhibitors (fluoxetine, fluvoxamine, paroxetine, sertraline)	62 (17.61)
Lithium	47 (13.35)
Sodium valproate, divalproex sodium	16 (4.54)
Diazepam	10 (2.84)
Amisulpride	9 (2.56)
Loxapine	6 (1.70)
Clozapine	5 (1.42)
Chlorpromazine	3 (0.85)
Alprazolam, clonazepam, donepezil, phenytoin, lorazepam, nitrazepam, risperidone	2 (0.57) each
Mirtazapine	1 (0.28)

The ADRs associated with olanzapine were weight gain (47), tremor (19), constipation (20), dyspepsia (10), amenorrhea (8), sedation (5), impaired glucose tolerance (2) and frank diabetes mellitus (1)

Some interesting ADRs were noted during the course of study. One case of olanzapine induced diabetes mellitus was noted in a 43-year-old female schizophrenic patient. She was on olanzapine therapy for last 2 years and developed symptoms of polyuria and polydipsia. She had no history of raised blood sugar prior to starting olanzapine; her fasting blood glucose level was 237 mg/dL at the time of the study but returned to normal on substitution of olanzapine with risperidone. We also found a rare case of risperidone induced rabbit syndrome, in a 34-year-old male schizophrenic patient. A case of donepezil induced painless blisters on face and forearm, after very first dose, was encountered in a 67-year-old male Alzheimer’s disease patient.

No ADR encountered turned out to be fatal, life-threatening or required hospitalization for management. Some of the events, such as tremor, were temporarily disabling but were managed by the clinicians with corrective medication (such as trihexiphenidyl for extrapyramidal symptoms) or dose modification.

## Discussion

The present study has reported the incidence and attempted to profile suspected ADRs to psychotropic drugs in the psychiatry OPD setting in the Indian context. In contrast to reports of ADR profiles of individual drugs, there is a dearth of pharmacovigilance profiling of psychotropic agents in general, not only in India but also worldwide. A Brazilian study, conducted in 2001, analyzed 219 notifications of suspected ADRs of psychoactive medicaments and incriminated antidepressants as the commonest group responsible for ADRs, followed by antipsychotics.[8] A Bulgarian study reported that the ADR frequency of individual psychotropic drugs studied is less than 1%.[9] A knowledge, attitude and practice based study conducted in Norway found that ADRs can be prevented by collecting reliable information about their frequencies and possible risk factors.[10] In our study, which is based on active surveillance rather than spontaneous reporting, we found antipsychotics to be most commonly responsible; this could be partly related to the frequency and duration of their use in the hospital setting.

Olanzapine was frequently prescribed in our setting, as it was dispensed, free of cost, from the hospital pharmacy. Although several new psychotropic drugs have been introduced in the Indian pharmaceutical market over the last few years (e.g., aripiprazole, quetiapine, reboxetine, venlafaxine, ziprasidone), they were not included in our report because they are relatively expensive and not dispensed from the hospital pharmacy. Hence, they were seldom prescribed in our setting – a public hospital catering mostly to economically weaker sections of society.

Regarding causality assessment, our study had no “certain” cases since the suspected ADRs were mostly of mild to moderate severity and hence did not require withdrawal of therapy. In cases where dechallenge was done, rechallenge was not attempted with the offending drug. This is in contrast to the Brazilian study where 24 cases were found to be “definite” after rechallenge was attempted.

Regarding the particularly interesting ADRs noted, olanzapine is known to cause metabolic syndrome[11] and so should be used with caution in schizophrenic patients requiring long-term therapy. Risperidone, an atypical antipsychotic, is known to produce tardive dyskinesia uncommonly,[12] but there are few reports on risperidone causing rabbit syndrome.[13] In our subject, perioral tremor was induced after risperidone was used at 4 mg/day for approximately 3 years. Donepezil, a reversible cholinesterase inhibitor, is rarely known to produce acute skin reactions.[14] In fact, we could not find any similar report of painless blister formation with donepezil.

Our study had limitations. For logistical reasons, we screened patients on two fixed days of each week, rather than rotating days, and this could introduce potential bias in the sample. Being an OPD-based study, it is likely that we have missed ADRs that were transient or too mild to have inconvenienced the patient to an extent sufficient to report to the doctor on the next hospital visit. Although routine hematological and clinical chemistry (e.g., blood sugar, lipids) reports were available, we could not generally order tests like ECG screening of patients for QT interval prolongation or blood sampling to determine serum prolactin concentration. There was no access to therapeutic drug monitoring (TDM) apart from lithium. However, though TDM of psychotropic agents has been employed, there is lack of consensus regarding its optimum use in clinical practice.[15]

Although this post-marketing surveillance study cannot provide true incidence or prevalence figures, it offers a representative idea of the ADR profile of psychotropic drugs likely to be encountered in ambulatory patients in an Indian public hospital. Compliance with therapy is a major issue in psychiatric patients. Constant vigil in detecting ADRs and subsequent dose adjustments can make therapy with psychotropic drugs safer and more effective. This, in turn, should improve compliance. ADRs can perhaps also be reduced by using less medication and with adequate knowledge of drug interactions. A psychotropic drug ADR database built up on the basis of such studies conducted across multiple centers, through active collaboration of psychiatrists and pharmacologists, can be a worthy long-term goal in the Indian context. Such a database can provide early warning signals of drug-reaction links if kept under active scrutiny

## References

[1] Aronson JK (2006). Risk perception in drug therapy. Br J Clin Pharmacol.

[2] Rani FA, Byrne PJ, Murray ML, Carter P, Wong IC (2009). Paediatric atypical antipsychotic monitoring safety (PAMS) study: Pilot study in children and adolescents in secondary- and tertiary-care settings. Drug Saf.

[3] Glassman AH, Bigger JJ (2001). Antipsychotic drugs, prolonged QTc interval, torsades de pointes and sudden death. Am J Psychiatry.

[4] Cooper C, Bebbington P, King M, Brugha T, Meltzer H, Bhugra D (2007). Why people don’t take their psychotropic drugs as prescribed: Results of the 2000 National Psychiatric Morbidity Survey. Acta Psychiatr Scand.

[5] Faich GA (1996). US adverse drug reaction surveillance 1984-1994. Pharmacoepidemiol Drug Saf.

[6] Bavdekar SB, Karande S (2006). National pharmacovigilance program. Indian Pediatr.

[7] (2005). The use of the WHO-UMC system for standardized case causality assessment [monograph on the Internet]. http://www.who-umc.org/graphics/4409.pdf.

[8] Carlini AE, Nappo AS (2003). The pharmacovigilance of psychoactive agents in Brazil. Rev Bras Psiquiatr.

[9] Dimitrova Z, Doma A, Petkova V, Getov I, Verkkunen E (2002). Psychotropic drugs in Bulgaria-frequency and risk of adverse drug reactions. Boll Chim Farm.

[10] Castberg I, Reimers A, Sandvik P, Aamo TO, Spiqset O (2006). Adverse drug reactions of antidepressants and antipsychotics: Experience, knowledge and attitudes among Norwegian psychiatrists. Nord J Psychiatry.

[11] Newcomer JW (2005). Second-generation (atypical) antipsychotics and metabolic effects: A comprehensive literature review. CNS Drugs.

[12] Corell CU, Leucht S, Kane JM (2004). Lower risk of tardive dyskinesia associated with second generation antipsychotics: A systemic review of 1 year studies. Am J Psychiatry.

[13] Ibrahim E, Ozcankaya R, Altinayazar V (2004). Risperidone-induced rabbit syndrome in mood disorder. Eur Psychiatry.

[14] (2006). ARICEPT^®^ (Donepezil Hydrochloride) package insert [monograph on the internet]. Woodcliff Lake.

[15] Baumann P, Heimke C, Ulrich S, Eckermann G, Gaertner I, Gerlach M (2004). The AGNP-TDM expert group consensus guidelines: Therapeutic drug monitoring in psychiatry. Pharmacopsychiatry.

